# Biological Responses of Onion-Shaped Carbon Nanoparticles

**DOI:** 10.3390/nano9071016

**Published:** 2019-07-15

**Authors:** Jaehee Jang, Youngjun Kim, Jangsun Hwang, Yonghyun Choi, Masayoshi Tanaka, Eunah Kang, Jonghoon Choi

**Affiliations:** 1 School of Integrative Engineering, Chung-Ang University, Seoul 06974, Korea; 2 School of Chemical Engineering & Material Science, Chung-Ang University, Seoul 06974, Korea; 3 Department of Chemical Science and Engineering, Tokyo Institute of Technology, Tokyo 152-8550, Japan

**Keywords:** carbon nanomaterial, nano-onion, biological properties, cytotoxicity, immune responses

## Abstract

Nanodiamonds are emerging as new nanoscale materials because of their chemical stability, excellent crystallinity, and unique optical properties. In this study, the structure of nanodiamonds was engineered to produce carbon nano-onion particles (CNOs) with multiple layers. Following a series of physicochemical characterizations of the CNOs, various evaluations for biological responses were conducted for potential biotechnological applications of the CNOs. The possibility of biological applications was first confirmed by assessment of toxicity to animal cells, evaluation of hemolysis reactions, and evaluation of reactive oxygen species. In addition, human immune cells were evaluated for any possible induction of an immune response by CNOs. Finally, the toxicity of CNOs to *Escherichia coli* present in the human colon was evaluated. CNOs have the chemical and physical properties to be a unique variety of carbon nanomaterials, and their toxicity to animal and human cells is sufficiently low that their biotechnological applications in the future are expected.

## 1. Introduction

Nanomedicine is broadly defined as the biomedical application of nanotechnology. More specifically, nanomaterials with a variety of physical, chemical, and biological characteristics can be employed in biomedical applications to overcome difficulties that have remained unresolved. Recently, new therapeutic and diagnostic methods have been developed by combining simple supramolecular components using nanotechnology [1,2,3,4,5]. Examples include the detection of pathogenic antigens [6], diagnosis and imaging of disease [7], development of drug delivery vehicles [8,9,10], and development of antibacterial agents [11]. Carbon nanomaterials with various dimensions, such as one-dimensional carbon nanotubes, two-dimensional graphene, and zero-dimensional fullerenes, are very attractive nanomaterials. They have gained much attention and have been actively studied because of their small nanoscale size and various physicochemical characteristics that are due to the large surface-to-volume ratio. For example, applications in tissue engineering [12,13], drug or gene transfer biosensors [14], photothermal therapy [15], and antibacterial substances [16] are actively being pursued. Recently, carbon nanomaterials with various new structures, such as carbon dots or nanodiamonds, have been developed and are being continuously studied. However, some carbon nanomaterials have been reported to be toxic to certain cells or animals and to induce an immune response [17,18,19]. Carbon nanomaterials within the mammalian cell are reported to promote reactive oxygen species (ROS), which are the cause of the toxicity. The incidence and toxicity of ROS are known to vary depending on the type of carbon nanomaterial [20]. Therefore, a thorough evaluation of biocompatibility in the use of carbon nanomaterials in the field of nanomedicine is inevitable. Carbon nanomaterials such as graphene, graphene oxide, carbon nanotubes (CNTs), and acid functionalized CNTs have been studied for their potential toxicity and their cytotoxic mechanisms in cells for further biomedical applications. It was found that neat graphene was toxic when treated with a Raw 264.7 cell by inducing the production of reactive oxygen species and apoptosis [21] and the nanocomposite of metal and graphene can be applied as an electrochemical sensor, using excellent thermal conductivity and electric conductivity [22]. Graphene oxide is involved in the release of lactate dehydrogenase (LDH) released from dead or damaged cells by apoptosis or necrosis and causing toxicity, due to the accumulation of autophagosome [23]. There are many applications for the application of graphene oxide, but, for example, there is a case applied to photodynamic therapy (PDT). By directly coupling the polyethylene glycol (PEG) with functional groups on the graphene oxide sheet, the dispersibility of the existing graphene oxide was further improved. Furthermore, branched polyethyleneimine (BPEI) was conjugated using EDC-NHS coupling. BPEI increased the loading capability of Chlorin e6 (Ce6), a photosensitizer, and increased photodynamic efficacy [24]. In addition, single-walled carbon nanotubes (SWCNTs) have been shown to cause damage to mitochondria [25], and the acid functionalized SWCNTs were also found to be involved in LDH release and accumulation in the autophagosome, leading to toxicity. [23]. Multi-walled carbon nanotubes (MWCNTs) likewise have been shown to induce apoptosis, the release of LDH [26], and mitochondrial damage [27]. The toxicity of SWCNTs, MWCNTs, and acid functionalized MWCNTs to intestinal microbes was evaluated and found to be antimicrobial. All of these can be applied as antimicrobial agents and CNTs have been reported to exhibit antibacterial effects by destroying the cell walls and membranes of bacteria [28]. Furthermore, carbon nanomaterials are used as a genosensor [29], drug delivery carrier [30], and biosensor [31]. Table 1 summarizes carbon materials for their main biological applications and toxicity.

Previous studies on properties and toxicity of carbon nano-onions (CNOs) have been carried out. A team of researchers studied surface functionalization to improve the problems of existing CNOs [32,33]. The other team evaluated the derivatives of CNO against *Hydra vulgaris* [34], providing a model system for the application of anti-microbial carbon nanomaterials. In this study, we synthesized CNOs (multi-walled fullerenes) using nanodiamonds. In addition, carboxyl groups were introduced on the surface of CNO to increase the water dispersion of the nanomaterials, which is essential for their biomedical applications. We investigated the feasibility of biomedical applications of CNOs by analyzing their physicochemical characteristics and biological responses. We evaluated whether CNO is a biocompatible carbon nanomaterial by performing assessments of CNO cytotoxicity to human dermal fibroblast (HDF) cells and peripheral blood mononuclear cells (PBMCs), immune response assays, hemolysis evaluations, assays for ROS generation (which is the main toxic factor of carbon nanomaterials), and measurement of toxicity to *Escherichia coli* intestinal bacteria. In conclusion, CNO showed no toxicity to HDF cells and PBMCs and did not induce the secretion of IL-2 and tumor necrosis factor-alpha (TNF-alpha) (which are cytokines in human immune cells). Moreover, the hemolysis rate tended to be very low, and CNO was not toxic to *E. coli*. Particularly because of the low occurrence of ROS, CNO is considered promising for use as a new carbon nanomaterial in research in various biomedical fields, such as tissue engineering, drug delivery systems, and biosensors.

## 2. Materials and Methods

### 2.1. Pyrolysis of Nanodiamond

Carbon nano-onions (CNOs) were generated by the pyrolysis of nanodiamonds (NDs) (Plasma Chem, Berlin, Germany) at high temperature. A crucible of ND (3 g of gray powder) was placed in a furnace tube and annealed at 1400 °C for 1 h under a nitrogen purge. The temperature was gradually lowered to room temperature under nitrogen. The NOs obtained in this annealing process were entirely black. 

### 2.2. Carboxylated Nano-Onion

**Figure 1:**
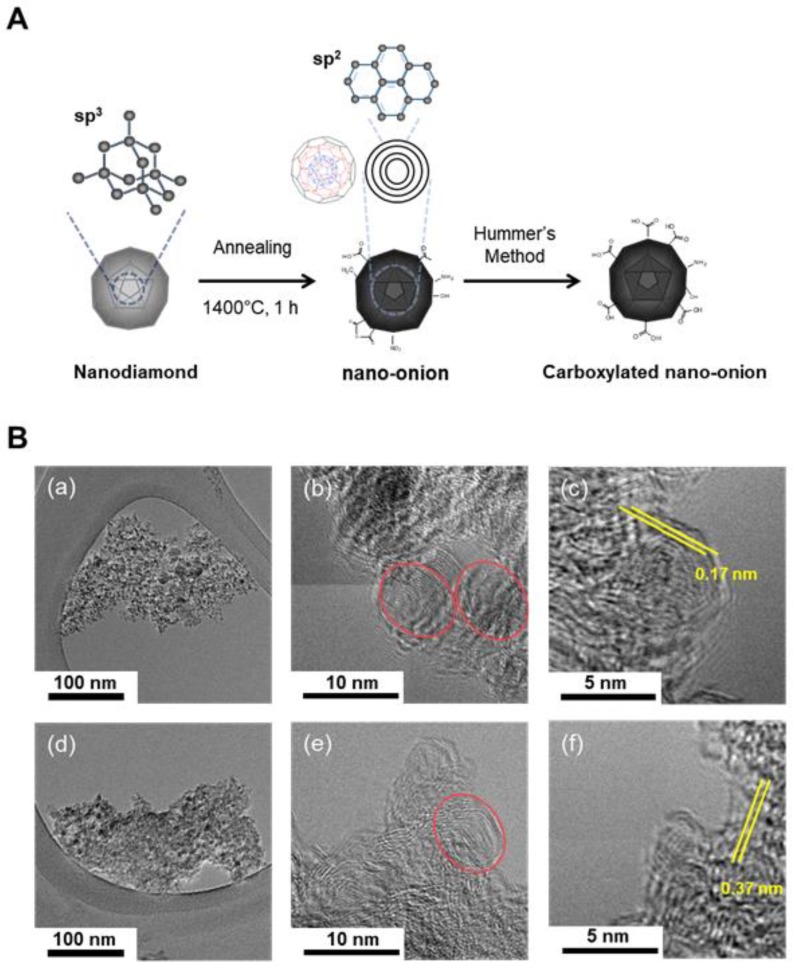
(**A**) Schematic drawing of structural changes from nanodiamond into carbon nano-onion particles (CNO) by pyrolysis and into CNO–COOH by Hummers’ method; (**B**(a–c)) Transmission electron microscopy (TEM) images of CNO, and (**B**(d–f)) CNO–COOH. The crystalline lattice gap of the onion-like layer is present in both CNO (B(c)) and CNO–COOH (B(f)), yellow bars showing the gap between lattices.

The surface of the CNO treated by pyrolysis was functionalized with carboxyl groups by the Hummers’ method [35] to create CNO–COOH, as shown in Figure 1. Sulfuric acid (360 mL) and phosphoric acid (40 mL) were carefully added to a round bottom flask in an ice bath. CNO (3 g) and potassium permanganate (9 g) were added into the sufficiently cooled sulfuric acid/phosphoric acid solution. The CNO was homogeneously dispersed for 1 h using a bath sonicator. The CNO dispersion was oxidized with gentle stirring at 50 °C. After 12 h, 800 mL of deionized (DI) water was added to the dispersion; the mixture was cooled and then combined with hydroperoxide (3 mL) to stop reactivity. The mixture was vacuum-filtered using a 0.2 μm polytetrafluoroethylene (PTFE) hydrophilic membrane filter (SciLab, Seoul, Korea). The filtered compact cake was washed twice with DI water (200 mL), HCl (200 mL), and ethanol (200 mL) through vacuum filtration. Finally, after washing with diethyl ether (200 mL), the cake was dried overnight in an 80 °C air-circulating oven.

### 2.3. Characterization

X-ray photoelectron spectroscopy (XPS) measurements were performed with a K-alpha+ spectrometer (Thermo Fisher Scientific, Waltham, MA, USA), using an Al Kα energy source. The spectra were analyzed using Advantage software. Transmission electron microscopy (TEM) images were acquired using a JEM-2100F electron microscope (JEOL, Tokyo, Japan). The CNO dispersion was dropped onto a Lacey Formvar/Carbon 200-mesh grid (Ted Pella, Redding, CA, USA) and dried for 10 min in a 60 °C oven. CNO and CNO–COOH(1 mg) were dispersed in ethanol (1 mL) and diluted with an appropriated concentration for the images.

### 2.4. Cell Viability Assay

Cell viability was determined using a CCK-8 cell counting kit [36] (Dojindo Molecular Technology, Kumamoto, Japan). Cells were seeded with equal density into each well of 96-well plates (5 × 10^3^ cells per well), using 100 µL of cell culture medium (low-glucose Dulbecco’s Modified Eagle Medium (DMEM), supplemented with 10% (v/v) fetal bovine serum and 1% sterile antibiotic), and were incubated for 24 h at 37 °C. Cells were then treated in 96-well plates with varying concentrations of CNO and CNO–COOH particles in a serum-free medium for 24 h at 37 °C. Untreated cells served as a control group. At the end of the treatment, CCK-8 dye was added to each well, and the plates were incubated for another 2 h at 37 °C. To prevent particles from interfering with this assay, the solution in each well of each plate was quantitatively transferred to an empty well in another plate after centrifugation. Subsequently, the absorbance was measured at 450 nm, using a microplate reader. Each treatment was repeated three times. The cell viability with the CNO and CNO–COOH was further assessed using a LIVE/DEAD^®^ Viability/Cytotoxicity Kit (Invitrogen™; Life Technologies, Carlsbad, CA, USA). The kit can quickly discriminate live from dead cells by simultaneously staining with green-fluorescent calcein acetoxymethyl ester to indicate intracellular esterase activity and with red-fluorescent ethidium homodimer-1 to indicate loss of plasma membrane integrity. After 24 h of incubation with varying concentrations of CNO and CNO–COOH, the culture medium was removed. Next, 200 µL of LIVE/DEAD stain was added to each well, and the wells were incubated for 30 min at 37 °C. Finally, the samples were observed using a fluorescence microscope.

### 2.5. Hemolysis Test

Aliquots (1 mL) of 2% red blood cells (from sheep blood) suspended in phosphate-buffered saline (PBS) were mixed with CNO and CNO–COOH solution (final concentrations of CNO and CNO–COOH were 5000, 10,000, 50,000, and 100,000 ng/mL each) and incubated at 37 °C for 1 h. The samples were then centrifuged at 2000 rpm for 5 min to remove intact red blood cells, and the absorbance of the supernatant was measured at 545 nm for the release of hemoglobin. PBS and 5% Triton X-100 were used as a negative and positive control, respectively. All measurements were performed in triplicate, and the hemolysis rate (%) was determined as HR (%) = (OD_sample_ − OD_negative control_)/(OD_positive control_ − OD_negative control_) × 100% [37].

### 2.6. Intracellular Reactive Oxygen Species Measurement

The intracellular ROS was determined using a well-characterized probe, namely 2ʹ,7ʹ-dichlorofluorescein diacetate (DCFH-DA) [38]. DCFH-DA passively enters the cell and is hydrolyzed by esterases to DCFH. This nonfluorescent molecule is then oxidized to the fluorescent compound dichlorofluorescein (DCF) by cellular oxidants. A 10 mM DCFH-DA stock solution (in methanol) was diluted 1000-fold in the cell culture medium without serum or other additives to yield a 10 mM working solution. Cells were washed twice with PBS and then incubated with DCFH-DA working solution for 20 min in a dark environment (37 °C incubator). This was followed by treatment with varying concentrations of CNO and CNO–COOH particles for 24 h. The cells were then washed three times with PBS to eliminate DCFH-DA that did not enter the cells. Cells were collected in suspension and the fluorescence was determined at 488 nm excitation and 525 nm emission, using a fluorescence spectrophotometer.

### 2.7. Cytokine Profiling Assay

The cytokine profiling was performed using the enzyme-linked immunosorbent assay (ELISA) [39]. An unlabeled capture antibody was diluted to a final concentration of 0.5–8 µg/mL in coating buffer (Cat. No. 421701, BioLegend, CA USA) and 100 µL were transferred to each well of a high-affinity, protein-binding ELISA plate (for example BioLegend Cat. No. 423501). The plate was incubated at 4 °C overnight. After three washes with PBS/Tween, non-specific binding sites were blocked by adding 200 µL of blocking solution to each well. The plate was incubated at room temperature for 1 h. After three additional washes with PBS/Tween, 100 mL of the supernatant of cells treated with varying concentrations of CNO and CNO–COOH particles was added to each well in the ELISA plate and incubated at room temperature for 2–4 hours. After another three washes with PBS/Tween, 100 mL of biotin-labeled detection antibody, diluted to a concentration of 0.25–2 µg/mL in blocking solution, was added to each well and incubated at room temperature for 1 h. After another three washes with PBS/Tween, 100 mL of Av-HRP conjugate (BioLegend Cat. No. 405103) at its predetermined optimal concentration in blocking buffer (usually between 1/500 and 1/2000) was added to each well. After incubation and washing, 100 mL of TMB Reagent was transferred to each well and incubated at room temperature for color development. The optical density (OD) of each well was read with a microplate reader at 450 nm wavelength.

### 2.8. Bacterial Tests

The in vitro bacterial activities of CNO and CNO–COOH were examined using the colony counting method [40]. Gram-negative *E. coli* (ATCC 25922) were used as microorganisms. Sterilized Luria–Bertani (LB) broth was measured (1 mL) into sterile tubes. The CNO and CNO–COOH at varying concentrations (1.5625, 3.125, 6.25, 12.5, 25, and 50 µg/mL) were introduced into the LB broth solution, which contained approximately 1.5 × $10^{5}$ colony forming units (CFU) of *E. coli*. The mixtures were cultured at 37 °C in a shaking incubator for 12 h. Pure PBS buffer and antibiotics were also tested as a negative control and positive control, respectively. A 100 µL aliquot of each of these cell solutions was seeded onto LB agar using a surface spread plate technique. The plates were incubated at 37 °C for 24 h. The bacterial CFUs were then counted to calculate the survivors.

### 2.9. Statistical Analysis

A one-tailed Mann–Whitney U test was performed using GraphPad Prism (v 7 for Mac OS X; GraphPad Software, La Jolla, CA, USA).

## 3. Results and Discussion

In this study, the sp^3^ structure nanodiamond was annealed at 1400 °C for 1 h, and then the sp^2^ structure CNO was synthesized. The CNO–COOH was subsequently synthesized by Hummers’ method (Figure 1A). The TEM images of CNO and CNO–COOH showed visualized features and the lattice gap between molecular layers, as shown in Figure 1B. Both CNO and CNO–COOH show the amorphous onion-like layers, unlike the crystalline structure of the nanodiamond. CNOs exist as aggregated forms between 300 and 400 nm, while each CNO is 5–8 nm. High-magnification TEM images of CNO and CNO–COOH show lattice gaps of 0.17 nm and 0.37 nm, respectively. The greater spacing in the lattice compared to the crystal has the potential to serve as a reservoir for electrons or small-molecule drugs. Two-dimensional graphene is believed to complex with small molecules via phi–phi stacking or physical adsorption, as small molecules settle in the interface between sheets.

The XPS spectra of the C1s and O1s peaks of the CNO and CNO–COOH are shown in Supplementary Materials Figure S1A. CNO consists mainly of carbon (the 97.96%) and has low contents of oxygen (1.47%) and nitrogen (0.57%). In contrast, a CNO–COOH has high oxygen content (19.16%), with 80.13% carbon and 0.71% nitrogen. A simple comparison shows that the oxygen content increased 13-fold in the transformation of CNO into CNO–COOH, indicating that oxidation by the Hummer’s method was functionalized into the carboxylation of the onion surface (Supplementary Materials Figures S1 and S2 and Supplementary Materials Table S1). The O1s peaks of the CNO and CNO–COOH were deconvoluted into three components at 531.6 eV (C=O) and 533.1 eV (C–OH and C–O–C). Based on the O1s peak, the relative C=O contents were 19.9 and 26.9% for the CNO and CNO–COOH, respectively (Supplementary Materials Table S2). The amplification of the C=O carbonyl group content and oxygen content indicates that the carboxyl group was successfully functionalized on the CNO surface. The C1s peaks of the CNO and CNO–COOH were deconvoluted into six component peaks at 284.6 (–C=C–), 285.4 (–C–C–), 286.0 (C–O), 287.2 (C=O), 289 (–COO–), and 290.55 eV (π–π*). The relative contents of –C=C–, which present an amorphous onion-like sp2 layer, were similar (54.0% and 49.7%) for the CNO and CNO–COOH. In contrast, the relative contents of –C=O– and –COO– were 18.13 and 9.66%, respectively, for the CNO–COOH, showing the high carboxylate content on the CNO surface.

**Figure 2:**
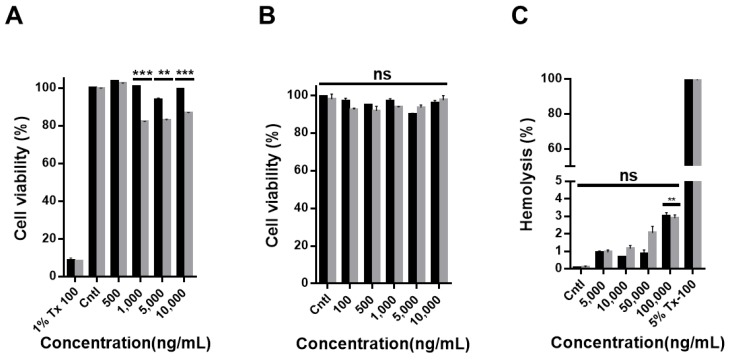
Cytotoxicity test of CNO (black bar) and CNO–COOH (gray bar) on human dermal fibroblast (HDF) cells and peripheral blood mononuclear cells (PBMCs). CCK-8 assay of treated samples incubated with varying concentrations (100, 500, 1000, 5000, and 10,000 ng/mL) of CNO and CNO–COOH with (**A**) HDF cells, and (**B**) PBMCs; (**C**) percent hemolysis of red blood cells incubated with varying concentrations (5000, 10,000, 50,000, and 100,000 ng/mL) of CNO and CNO–COOH.

HDF cells and human PBMCs, which are composed of lymphocytes (for example T cells, B cells, and NK cells) and monocytes, were used to determine the biocompatibility of CNO and CNO–COOH (Figure 2). Cytotoxicity tests were performed by using the CCK-8 assay after 24 h of sample treatment with different concentrations (namely 100, 500, 1000, 5000, and 10,000 ng/mL). For the negative control, the basal medium without any nanoparticle sample was added to the cells. As a positive control, Triton X-100 was used, which is known to dissolve the cell membrane as a surfactant and to kill the cell. Results show that cell viability of the CNO treatment group is high at all concentrations, whereas CNO–COOH decreases the cell viability to 80% at particle concentrations higher than 1000 ng/mL (Supplementary Materials Figure S3A). In the image analyses of the HDF cells, an increased number of aggregated CNO particles are observed compared with CNO–COOH, because the number of ionizable functional groups are lower in CNO. Particle aggregation and hydrophobicity would be the primary reasons for CNO having lower toxicity than CNO–COOH at the same concentration when co-cultured with cells. This phenomenon is also common in other carbon nanomaterials. In a previous report [41], a comparison of the toxicity of reduced graphene oxide with that of a neat graphene oxide showed that the reduced graphene oxide, which has better solubility in an aqueous environment, was highly toxic. The cell viability test with PBMC also showed no toxicity with either CNO or CNO–COOH at any concentration (Supplementary Materials Figure S3B). It should also be noted that CNO is present as an aggregate due to lack of oxygen function and is larger than CNO–COOH. CNO–COOH has excellent dispersibility due to its large number of oxygen functional groups. As CNO–COOH exists as individual particles, it has a large surface area to contact with cells and is more cytotoxic than CNO.

Hemolysis, an important consideration for the blood compatibility of nanoparticles, was tested with both CNO and CNO–COOH by co-culturing them with red blood cells. The test consisted of measuring the concentration of hemoglobin leaking out of red blood cells because of the collapse of the red blood cells. As shown in Figure 2C, both CNO and CNO–COOH tended to increase the rate of hemolysis in proportion to their concentrations, but showed a hemolysis rate of less than 4%, even at a very high particle concentration of 100,000 ng/mL.

**Figure 3:**
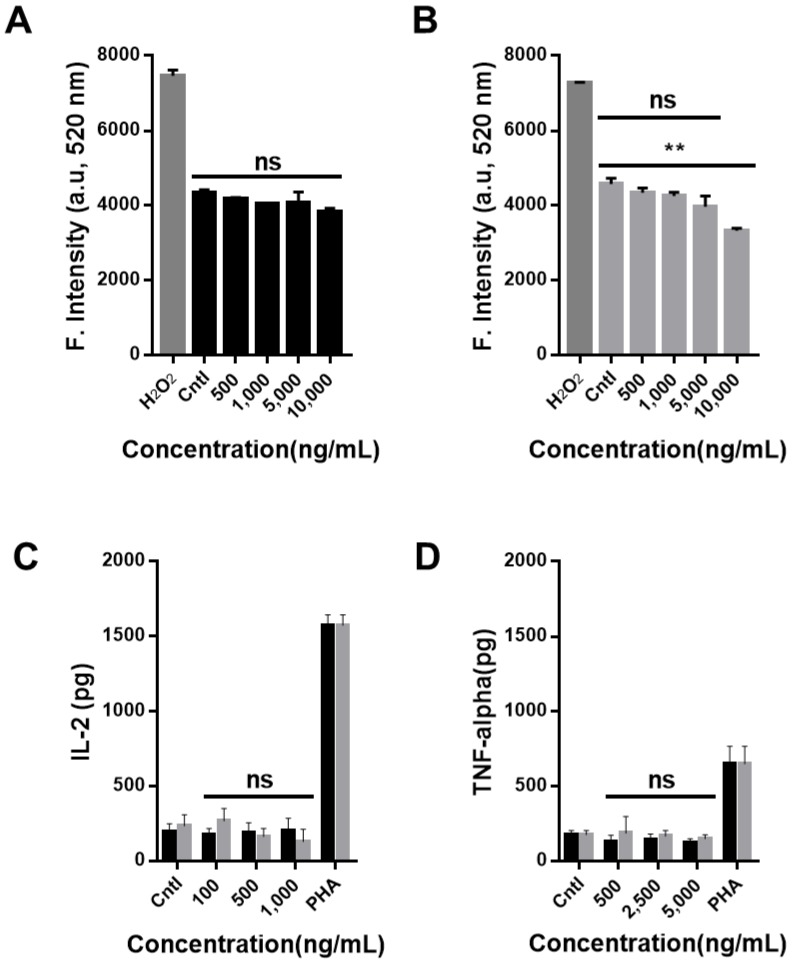
Fluorescence intensity according to the quantity of reactive oxygen species (ROS) of DCFDA (2’,7’ –dichlorofluorescin diacetate) on HDF cells after sample treatment with varying concentrations (500, 1000, 5000, and 10,000 ng/mL) for (**A**) CNO (black bar), and (**B**) CNO–COOH (gray bar); (**C**) production of IL-2 after sample treatment on PBMCs with varying concentrations (100, 500, and 1000 ng/mL); (**D**) production of tumor necrosis factor-alpha (TNF-alpha) after sample treatment on PBMCs with varying concentrations (500, 2500, and 5000 ng/mL).

ROSs are highly reactive molecules containing oxygen ions and hydrogen peroxide. Oxidative stresses, due to their high reactivity, can damage the cell structure. ROS generation caused by the presence of carbon-based nanomaterials is one of the major causes of induced cell cytotoxicity. Therefore, the quantity of ROS produced at various concentrations (500, 1000, 5000, and 10,000 ng/mL) of CNO and CNO–COOH was investigated (Figure 3). The negative control was treated with a neat basal medium, and the positive control was treated with hydrogen peroxide as reactive oxygen species. The results show that a substantial quantity of ROS was detected in the positive group treated with hydrogen peroxide, and no significant differences were found among the CNO, CNO–COOH, and control group; however, the ROS production decreased slightly with increasing concentrations of 500 to ~10,000 ng/mL. The quantity of ROS was reduced as the particle concentration increased because the quantities of CNO and CNO–COOH internalized in the cells also increased. CNO and CNO–COOH taken up in the cell would interfere with the ROS assay, quenching the fluorescence signal of ROS. This trend is more significant with CNO–COOH because of its excellent solubility and efficient cell penetration. It should be noted that the current assay results are correct for the CNO functionalized by Hummers’ method, having COOH, CHO, or C=O groups due to the oxidation processes. The ROS results here may not be generalized for all functionalized CNO.

The T cells in PBMCs secrete cytokines, induce proliferation of macrophages, and promote immune cell differentiation. To investigate the immunological responses of T cells possibly induced by CNO and CNO–COOH, the levels of interleukin-2 (IL-2) and tumor necrosis factor-alpha (TNF-alpha) secreted by the PBMCs were assayed using ELISA, with varying concentrations of CNO and CNO–COOH (Figure 3C–D). The results indicate that the quantity of IL-2 secreted by CNO and CNO–COOH was less than 500 pg in the negative control and at all concentrations of CNO and CNO–COOH. In addition, the quantity of TNF-alpha secreted was less than 200 pg in the negative control and at all concentrations of CNO and CNO–COOH. Therefore, CNO and CNO–COOH are negligibly toxic to human cells and non-immunogenic and are potentially biocompatible nanomaterials.

**Figure 4:**
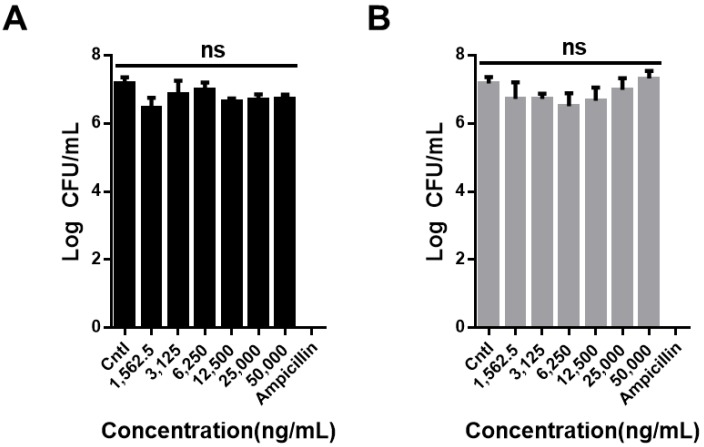
In vitro bacteria viability test of (**A**) CNO (black bar) and (**B**) CNO–COOH (gray bar) with varying concentrations (1562.5, 3125, 6250, 12,500, 25,000, and 50,000 ng/mL) using colony counting assay against *Escherichia coli.*

There are approximately 100 trillion microorganisms in the human body called human microbiota; this number is ten times higher than the number of human cells and includes both beneficial and harmful bacteria. These microorganisms are present in various parts of the body, such as the skin, oral cavity, genitalia, respiratory tract, and gastrointestinal tract. The gastrointestinal tract has the most numerous and greatest variety of microorganisms. The significant role that microorganisms play in interactions with the human body, such as absorption and metabolism of nutrients in the human body, maturation, development of the immune system and nervous system, and the occurrence and prevention of various diseases, is well documented. An upset in the balance between beneficial and harmful bacteria could lead to multiple diseases, such as obesity, diabetes, and colorectal cancer [42,43,44]. To test the cytotoxicity of CNO and CNO–COOH against microorganisms, *E. coli* was chosen as a model in our evaluation. The negative control was supplemented with the no-treatment medium, whereas the positive control was given an antibiotic-antimycotic. The test results showed that all the bacteria were killed in the positive control with antibiotics, but there was no difference between the negative control and CNO and CNO–COOH, even at their highest concentration of 50,000 ng/mL, and they did not affect the *E. coli* (Figure 4).

## 4. Summary

To evaluate the biocompatibility of CNO and CNO–COOH, an in vitro cytotoxicity evaluation, immunological assays, hemolysis test, ROS production analysis, and toxicity evaluation against *E. coli* were performed. CNO and CNO–COOH showed no toxicity to human HDF cells and PBMCs at concentrations below 500 ng/mL. Neither CNO induced IL-2 and TNF-alpha secretion significantly. The hemolysis rate was also low, indicating that CNO and CNO–COOH have blood compatibility. Neither CNO was toxic to the *E. coli* intestinal bacteria. The results of this study show that CNO and CNO–COOH have excellent biocompatibility because of the low occurrence of ROS, which is believed to be the leading cause of carbon nanomaterial toxicity. CNOs are promising for future use in diverse biomedical and biomolecular engineering applications, including drug delivery, theranostics, and biosensors. In this study, conjugation of the various biomolecule is possible by functionalizing COOH in CNO. For example, peptide, DNA, and protein can be attached to the COOH of carbon nanomaterials through EDC-NHS crosslinkers. A fluorescent dye, metal nanoparticles, or other non-biomolecular materials are also expected to be conjugated to COOH of carbon nanomaterials for further applications.

## Figures and Tables

**Table 1:** Toxicity and application of carbon nanomaterials.

Nanomaterial	Target Cell	Effect in Cells	Application	Ref.
Neat graphene	RAW 264.7	ROS generation, apoptosis induction	Electrochemical sensing	[21,22]
Graphene oxides (GO)	Peritoneal macrophages	LDH release, Autophagosome accumulation,	Photodynamic therapy (PDT)	[23,24]
SWCNTs	Mouse peritoneal macrophages	Mitochondrial damage	Antibacterial material	[25,28]
Acid functionalized SWCNTs	Peritoneal macrophages	LDH release, Autophagosome accumulation	Drug delivery carrier	[23,30]
MWCNTs	RAW264.7, A549	LDH release and oxidative stress	Genosensor	[26,29]
Acid functionalized MWCNTs	RAW 264.7	Apoptosis via the mitochondrial pathway	Biosensor	[27,31]

## Supplementary Materials

The following are available online at https://www.mdpi.com/2079-4991/9/7/1016/s1. **Figure S1.** XPS spectra of C1s (A and C) and O1s (B and D) peaks of CNO (A and B) and CNO–COOH (C and D). **Figure S2.** Spectral survey of (A) CNO and (B) CNO–COOH, showing increased oxygen contents. **Figure S3.** (A) Fluorescent microscopic images from live and dead assay for CNO and CNO–COOH with different concentrations on HDF cells after 24 h incubation; (B) microscopic images of PBMC after treatment of CNO and CNO–COOH with different concentrations and after 24 h incubation. **Table S1.** Atomic composition of CNO and CNO–COOH. **Table S2**. Components of CNO and CNO–COOH from C1s and O1s peaks.
