# Supplementary material for: Biological Responses of Onion-Shaped Carbon Nanoparticles
Source: Nanomaterials (Basel). 2019 Jul 15;9(7):1016. doi: 10.3390/nano9071016 (PMC6669643; doi:10.3390/nano9071016)
Supplement: Supplementary file 1 [file nanomaterials-09-01016-s001.pdf]

Supplementary Materials

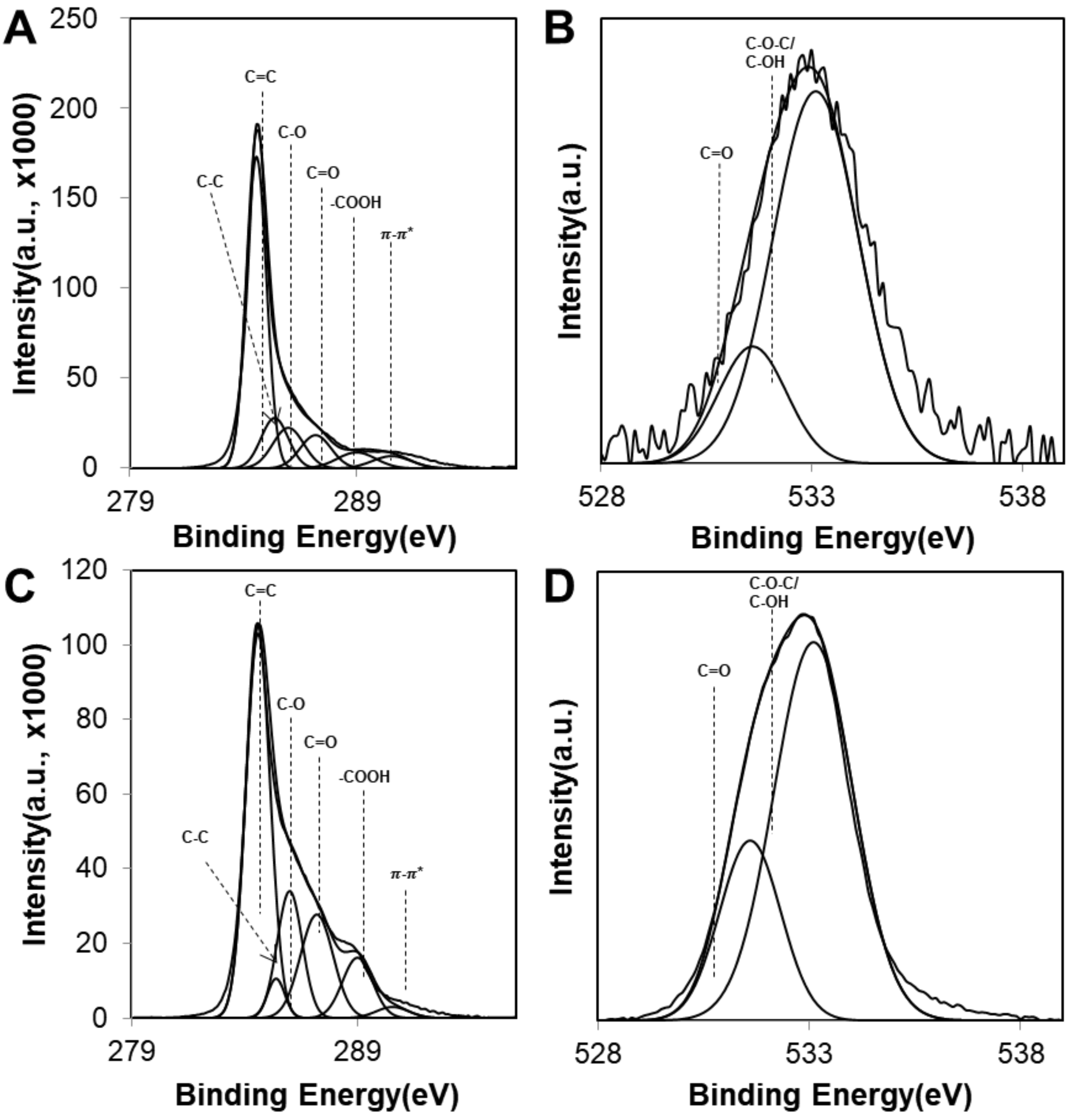

Figure S1. XPS spectra of C1s (A and C) and O1s (B and D) peaks of nano-onion (A and B) and carboxylated nano-onion (C and D).

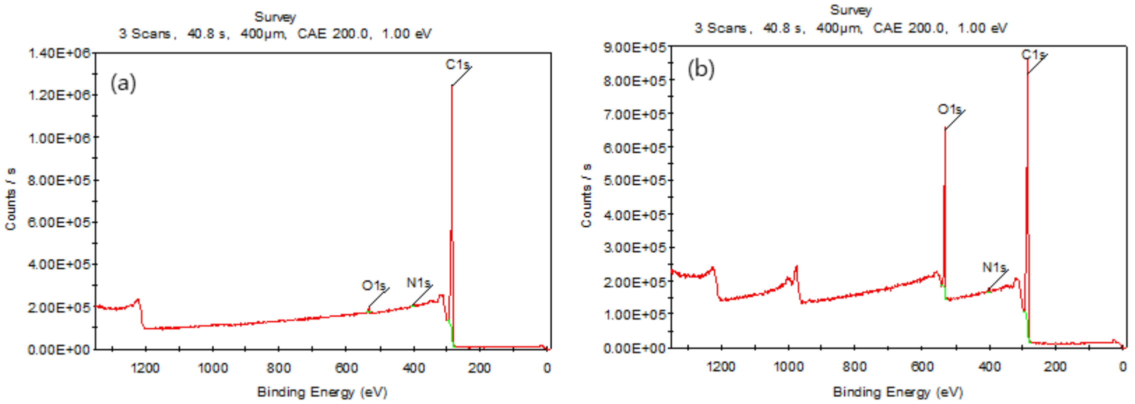

Figure S2. Spectral survey of (a) CNO(p) and (b) CNO–COOH, showing increased oxygen contents.

Table S1. Atomic composition of CNO(p) and CNO-COOH**.**

Atomic %	Carbon	Oxygen	Nitrogen
NO(p)	97.96	1.47	0.57
NO-COOH	80.13	19.16	0.71

Table S2. Components of CNO(p) and CNO-COOH from C1s and O1s peaks.

C1s(%)	NO(p)	NO-COOH
C=C (284.6 eV)	53.96	47.70
C-C (285.4 eV)	17.28	3.67
C-O (286 eV)	11.20	16.89
C=O (287.2 eV)	8.59	18.13
-COOH (289eV)	4.69	9.66
π-π* (290.55 eV)	4.27	1.94
**O1s(%)**		
C=O (531.6 eV)	19.9	26.9
C-O-C/C-OH (533.1 eV)	80.1	73.1

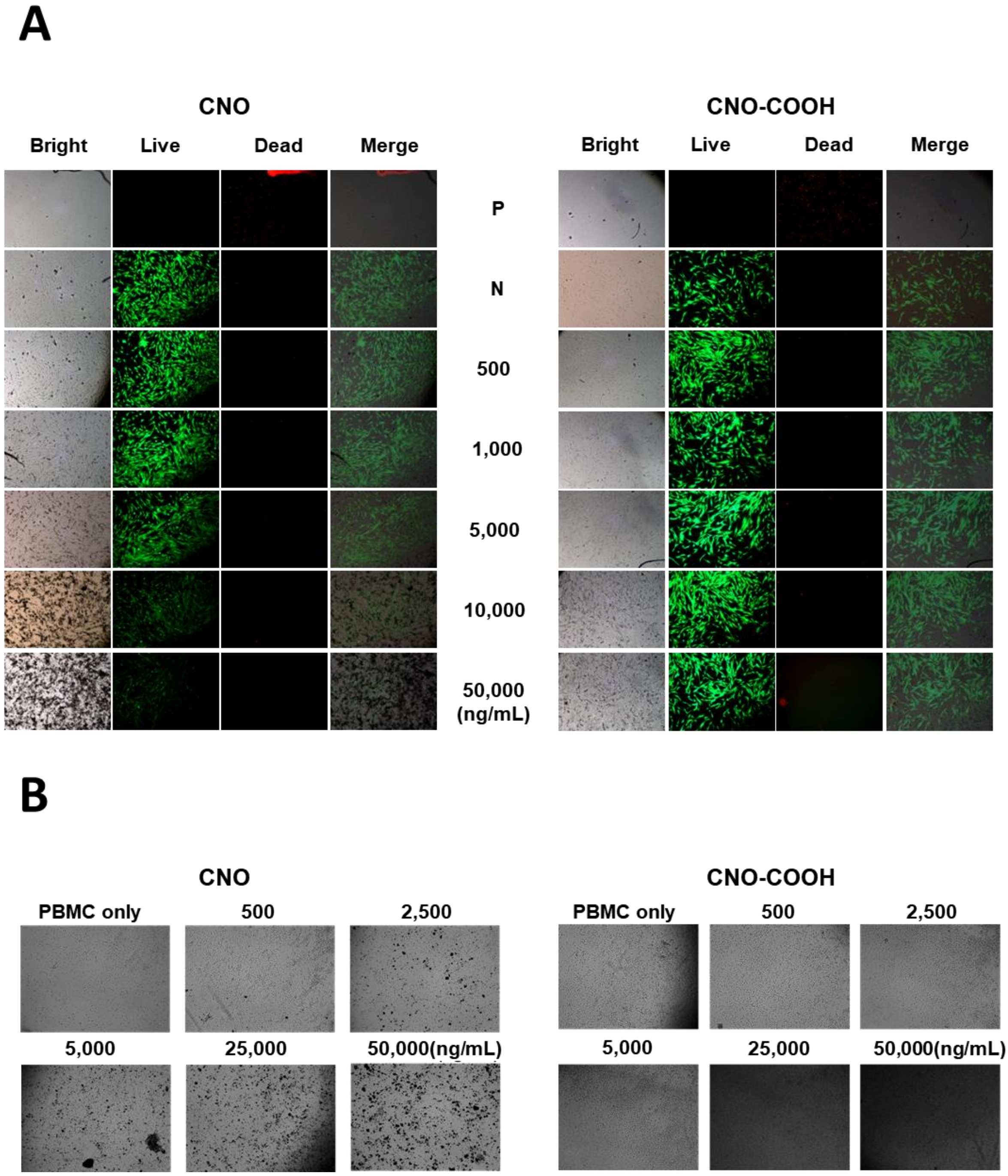

Figure S3. (A) Fluorescent microscopic images from live and dead assay for CNO and CNO–COOH with different concentrations on HDF cells after 24 h incubation; (B) microscopic images of PBMC after treatment of CNO and CNO–COOH with different concentrations and after 24 h incubation.
